# Social and Structural Determinants of HIV Treatment and Care Among Hispanic Women and Latinas Living with HIV Infection in the United States: A Qualitative Review: 2008–2018

**DOI:** 10.1089/heq.2019.0039

**Published:** 2019-11-04

**Authors:** Angelica Geter Fugerson, Madeline Y. Sutton, Donna Hubbard McCree

**Affiliations:** ^1^ Oak Ridge Institute for Science and Education, Oak Ridge, Tennessee.; ^2^ Division of HIV/AIDS Prevention, Centers for Disease Control and Prevention, Atlanta, Georgia.; ^3^ Department of Obstetrics and Gynecology, Morehouse School of Medicine, Atlanta, Georgia.

**Keywords:** HIV, Hispanic/Latino, women, HIV-positive persons, HIV disparities

## Abstract

**Purpose:** In 2017, among all women in the United States, Hispanic women and Latinas (Hispanics/Latinas) accounted for 16% of women with HIV. Populations with high HIV disparities, including Hispanics/Latinas, experience treatment and care outcomes that are well below the national goals. The objective of this qualitative review was to identify social and structural barriers to HIV care from the perspective of Hispanics/Latinas.

**Methods:** Our qualitative review was conducted in six stages: (1) searched and reviewed studies with a focus on Hispanics/Latinas with diagnosed HIV in the United States, published between January 2008 and August 2018; (2) removed unpublished reports and dissertations; (3) limited the search to keywords linked to social and structural HIV outcomes; (4) limited our search to studies that included samples of ≥30% Hispanics/Latinos and ≥30% female; (5) extracted and summarized the data; and (6) conducted a contextual review to identify common themes.

**Results:** We identified 1796 articles; 84 titles and abstracts were screened for full-text review; 16 were selected for full review; and 6 articles met our inclusion criteria for final analysis. Barrier themes to HIV care for Hispanics/Latinas included HIV-related stigma from health professionals, legal consequences of seeking HIV services (including fear of deportation), and language barriers while utilizing HIV services and medications.

**Conclusion:** Although the evidence addressing facilitators and barriers to care among HIV-positive women is sparse, interventions, resources, and enhanced training for health professionals to decrease social and structural barriers to HIV services for Hispanics/Latinas are warranted.

## Introduction

The benefits of HIV treatment to improving health outcomes among persons with HIV and reducing HIV transmission to persons at risk for infection are well documented.^1–4^ Persons with HIV who adhere to treatment as prescribed and have an undetectable viral load have effectively no risk of sexually transmitting HIV to their HIV-negative sex partners.^3–5^ Given the importance of HIV treatment to prevention efforts, reducing disparities in HIV treatment and care is a national goal.^6^ The goals to be achieved by 2020 for persons with diagnosed HIV infection include the following: (1) 85% linked to care within 1 month; (2) 90% retained in care; and (3) 80% with a suppressed viral load.^6^ The goals are further supported by the national Ending the HIV Epidemic plan to reduce new cases of HIV, many of which occur through transmission of persons who are unaware of their HIV-positive status or HIV-positive persons who are not in treatment or care.^7^ Populations with high HIV disparities (i.e., viral suppression by race/ethnicity), including Hispanic women/Latinas (hereafter referred to as Hispanics/Latinas), experience treatment and care outcomes that are well below the national goals.^8,9^

Hispanics/Latinos, who comprise about 18% of the U.S. population, accounted for 25% of HIV diagnoses in 2017 and about 24% of persons with HIV at the end of 2016.^10^ Of new HIV diagnoses in 2017, 19% were among females; the rate of new diagnoses among Hispanic/Latino females in 2017 (5.0) was three times the rate among white females (1.7).^10^ Among Hispanics/Latinos aged ≥13 years living with diagnosed HIV infection at the end of 2015, 71% were in receipt of care (≥1 test [CD4 or VL]), 58% were retained in care (≥2 tests [CD4 or VL] ≥3 months apart in 2015), and 60% were virally suppressed (<200 copies/mL on the most recent VL test in 2015).^10^ Furthermore, results from a recent analysis of treatment and care outcomes among Hispanics/Latinas aged ≥13 years with HIV infection showed that 63% were retained in care and 58% achieved viral suppression.^11^

The HIV-related disparities among Hispanics/Latinas are associated with myriad social and economic factors. Low rates of insurance,^12–14^ lower educational attainment,^15^ financial instability,^16^ and fear of deportation and detainment^17^ may prevent Hispanics/Latinas from seeking HIV testing and prevention services. Furthermore, fear due to undocumented immigration status, deportation, distrust of providers, and language barriers may prevent Hispanics/Latinas from accessing health care systems.^18^ There is a paucity of literature regarding factors associated with HIV treatment and care among Hispanics/Latinas with HIV (HLWH); therefore, the purpose of this qualitative review is to identify possible social and structural factors related to the disparities in treatment and care from the perspective of HLWH in the United States.

## Methods

**FIG. 1.**
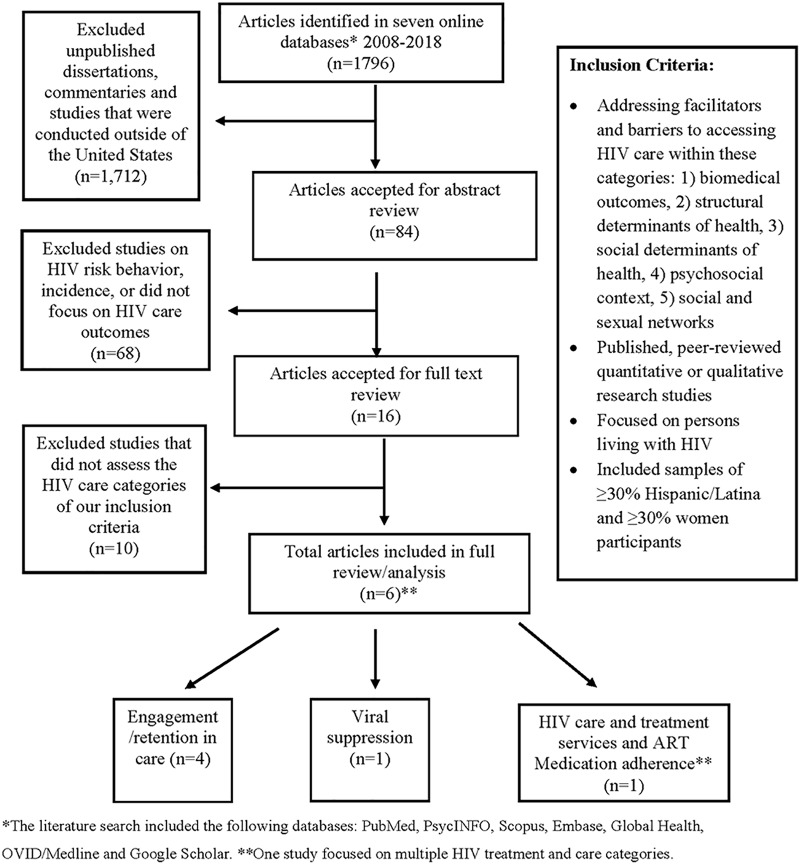
Selection process for qualitative review of the literature, HIV care outcomes among Hispanic Women/Latinas living with diagnosed HIV infection, 2008–2018. *The literature search included the following databases: PubMed, PsycINFO, Scopus, Embase, Global Health, OVID/Medline, and Google Scholar. **One study focused on multiple HIV treatment and care categories. ART, antiretroviral therapy.

The qualitative review was conducted in six stages using PubMed, PsycINFO, Scopus, Embase, Global Health, OVID/Medline, and Google Scholar. First, we searched for studies that included HLWH published between January 2008 and August 2018. Second, we excluded abstracts, unpublished dissertations, editorials, commentaries, and studies that were conducted outside of the United States. Third, we limited the results to five areas of inquiry regarding HIV treatment and care disparities and inequities among Hispanics/Latinas: (1) biomedical (care, treatment, antiretroviral therapy [ART], medication adherence, and viral suppression); (2) structural determinants of health (access to HIV care, patient/provider communication, and quality of HIV care); (3) social determinants of health (SDH) (stigma, discrimination, and medical distrust); (4) psychosocial context (peer support and mental health); and (5) social and sexual networks (social support and partner characteristics).^19^ Fourth, we focused on studies that had majority representation of HLWH in the United States, by requiring that the study population was ≥30% Hispanic/Latino and ≥30% female—the criteria were selected to ensure a representative sample of Hispanics/Latinas. Published quantitative and qualitative research studies that met the above inclusion criteria were included in the full-text review (Fig. 1). Fifth, we extracted and summarized these data in a table that highlighted the author names, year, location, study design, sample size, age, HIV continuum of care category, and major findings (Table 1).^19,20^ Sixth, we reviewed the articles and utilized direct content and themed analysis to identify common themes for barriers and facilitators for HIV treatment and care (Table 2).^21^ This study was reviewed and approved by the Centers for Disease Control and Prevention's institutional review and project determination process.

**Table 1.** Literature Review Findings of Social and Structural Barriers and Facilitators Contributing to HIV Treatment and Care Disparities Among Hispanics/Latinas Living with Diagnosed HIV Infection

First author	Source of the data, location	Study population	*N*, % Hispanic/Latina, % female, age mean years (SD) or (range)	Methodology	HIV care continuum category	Major findings
Dang et al.^26^	Primary data collection, Southern United States	Patients of HIV clinics; clients of social services agencies	*N*=22; 100% Hispanic/Latino, 59% female, mean age=39 (SD=8.9)	Qualitative: in-depth, face-to-face semistructured interviews	Engagement and retention in care	Barriers to engaging HIV services included fear of deportation and job loss, limited or no access to appropriate documentation to use HIV services, and HIV-related stigma from health care professionals and social networks.
Eaton et al.^22^	Primary data collection, southeastern United States	Patients of an HIV care clinic	*N*=28; 42% Hispanic/Latino; 39% female; mean age=43 (SD=N/A)	Qualitative; nominal group technique; multivoting analysis to quantify qualitative responses	ART medication adherence; HIV care and treatment services	Hispanics/Latinas were more concerned about access to HIV services, insurance coverage, out-of-pocket costs, and potential for disruption in HIV regimen due to deportation or end of short-term visa. Hispanic/Latinas expressed an interest in long-acting ART regimens and regimens coformulated with other medications (vitamins and supplements) to improve their quality of life.
Enriquez et al.^25^	Primary data collection, midwestern United States	Patients of three HIV care clinics	*N*=18; 100% Hispanic/Latino; 100% female; mean (N/A) (range=22–55)	Qualitative; one-on-one interviews	Engagement and retention in care	Barriers to engaging HIV services, health professionals, and continuity of HIV care included isolation, lack of social support, HIV-related stigma, language barriers/limitations, and medical mistrust.
Levison et al.^24^	Primary data collection, northeastern, United States	Patients of an HIV and primary care clinic and community organizations	*N*=51; 100% Hispanic/Latino; 32% female; mean age >26 years	Qualitative; semistructured interviews	Engagement and retention in care	Barriers to retention and engagement in HIV care included HIV-related stigma; avoidance of HIV care as a response to stigma; legal consequences such as deportation
Martinez et al.^23^	Primary data collection, midwestern and northeastern United States and U.S. territories	Patients from the Adolescent Medicine Trials Networks	*N*=14; 100% Hispanic/Latino; 43% female; mean age=21.5 (SD=2.2)	Focus groups and one-to-one interviews	Engagement/retention in care	HIV-related stressors included psychological responses (e.g., depression and anxiety), HIV-related stigma, physiological changes; medication side effects
McFall, et al.^27^	Primary data collection, northeastern United States and western United States	Participants of the Women's Interagency HIV Study	*N*=150; 46% Hispanic/Latino; 100% female; mean age=45.1	Structured interviews; physical examinations; biological specimens	Viral suppression	Predictors of not achieving viral suppression among Hispanics/Latinas included social and structural determinants of equity such as low annual household income, lack of health insurance, history of hepatitis C, and limited or nonparticipation in an AIDS Drug Assistance program.

**Table 2.** Emergent Themes for Barriers to HIV Care and Treatment Among Hispanics/Latinas Living with Diagnosed HIV Infection, 2008–2018

Lack of social support
Lack of insurance coverage
Out-of-pocket fees
Fear of legal consequences such as deportation
Limited employment opportunities and economic stability
Limited access to documentation to seek HIV services
Mental health outcomes such as depression and anxiety
HIV-related stigma from health care professionals
Language barriers and limitations when seeking HIV services

## Results

We identified 1796 articles; 84 relevant titles and abstracts were screened for full-text review, 16 were selected for full review, and 6 articles met inclusion criteria for final analysis (Fig. 1). The six studies were conducted throughout the United States (some studies were conducted in multiple cities throughout the United States), including the Northeast (*n*=3), Southeast (*n*=2), Midwest (*n*=3), and West (*n*=1) (Table 1). Barriers to improving the HIV care continuum among HLWH emerged during the review and are summarized in Table 2.

### Access to HIV care and treatment services

One study examined the structural determinants of access to HIV treatment and care services. Although most of the participants discussed topics such as viral control, improved CD4 counts, and quality of life, we highlighted results that were specific to Hispanics/Latinas. These responses included barriers to utilizing HIV services such as insurance coverage, out-of-pocket costs and problems related to use of short-term visas.^22^

### Engagement/retention in care

Four studies examined engagement and retention in HIV care among HLWH. During these studies, the women discussed social barriers to engagement and retention in HIV care, including HIV-related stressors such as depression, anxiety, medication side effects, and physiological changes due to the ART treatment,^23^ HIV-related stigma, avoidance of HIV care as a response to stigma, and legal consequences such as deportation.^24^ Furthermore, the women discussed social barriers to communicating with health professionals and continuity of HIV care. These factors included isolation, lack of social support, HIV-related stigma, language barriers and limitations, and medical distrust.^25^ The women also highlighted structural barriers to engaging HIV services, including potential deportation, work and limited access to time off and vacation days, and limited or no access to appropriate documentation for accessing services.^26^

### ART medication adherence

One study focused on multiple topics, including ART medication adherence. HLWH expressed an interest in pharmacological innovations to improve ART medication adherence, including ART medications administered by injection or long-acting pills that can be taken on a monthly basis. They also expressed a desire for ART medication coformulated with other medications (e.g., supplements and vitamins) to improve their quality of life.^22^

### Viral suppression

One study examined social and structural determinants of viral suppression. Among Hispanics/Latinas, 31% were virally suppressed and 28% had viral failure (not virally suppressed). Facilitators of viral suppression were not documented. Predictors of viral failure among Hispanics/Latinas included social and structural determinants of equity such as low annual household income, lack of health insurance, history of hepatitis C, and limited or nonparticipation in an AIDS Drug Assistance program (ADAP).^27^

## Discussion

Our review identified groups of studies related to ART medication adherence, engagement/retention in care, HIV care and treatment services, and viral suppression for Hispanics/Latinas diagnosed with HIV infection. These studies focused on themes that described barriers to HIV care and treatment, including HIV-related stigma from health professionals, lack of social support, legal consequences of seeking HIV services (including fear of deportation), language barriers while utilizing HIV services, and medical mistrust. These social and structural barriers are important to consider as interventions are developed and resources are distributed to improve care outcomes for HLWH.^28^

Regarding medication adherence, Hispanics/Latinas in one study indicated that longer acting formulations of ART medications would improve adherence and their quality of life. Longer acting ART formulation could also potentially address issues such as risk for treatment interruptions and lost to follow-up found in some Hispanic/Latino communities.^29^ Current trials of long-acting, injectable ART formulations are encouraging and show early efficacy.^30^ However, Hispanics/Latinos are underrepresented as defined participants in these trials.^31^ Enrolling Hispanics/Latinos in ART trials will help ensure the findings are relevant and applicable for these groups as part of global HIV prevention and care strategies.

The studies that examined engagement/retention in HIV care and treatment services described several political, legal, clinical, and political barriers for HLWH. These barriers, disproportionate effects of poverty, low health literacy, lack of access to high-quality care,^32^ and acculturation challenges,^33^ are aligned with other reports of SDH challenges in HIV treatment and care for HLWH. One report of strategies used by providers to engage and retain HLWH in care described multilevel social and structural techniques, including hiring Spanish-speaking staff and interpreters familiar with Latino culture, creating HIV ambulatory outpatient care centers with multiple specialties present on site, and offering supportive social services with flexible scheduling and Spanish-language materials.^34^ These approaches may be particularly effective for addressing acculturation challenges. In addition, programs such as Latinos in the Deep South, coordinated by the Latino Commission on AIDS, are seeking to build local leadership capacity and provide public health, advocacy, legal aid, and community-based participatory research support for Latinos affected by HIV in the South.^35^ The impact of these programs on HIV care for Hispanics/Latinas should be evaluated, and, if effective, replicated in other regions.^36^

In our review, barriers to reaching optimal viral suppression among HLWH also included SDH factors, such as low income, lack of health insurance, and no access to ADAP. While there is a dearth of reports specifically for HLWH, a report with mostly black women and Hispanics/Latinas also found SDH factors as barriers to viral suppression. Results suggested that women who are caring for children and are uncertain about wanting HIV care might need more intense monitoring and active follow-up.^37^ Maintaining ADAP, which is affected by national- and state-level fiscal and policy fluctuations, may play an important role in providing access, monitoring, and active follow-up for all persons with HIV,^38^ especially HLWH.

### Limitations

This qualitative review has limitations. First, five of six (83%) studies included sample sizes of only 14–51 women; one study enrolled 887 Hispanic/Latinas. Larger samples are needed in future studies to provide for analyses that are more robust and improve the generalizability of the results. Second, geographic location may play a role in the context of facilitators and barriers to health care for Hispanics/Latinas. In recent years, this is especially true in the southern United States, an area that has noted more new HIV diagnoses and new sociopolitical challenges for all Hispanic/Latino subgroups than any other U.S. region. As we study and better understand these potential regional sociopolitical factors, we can better account for them in intervention development for HLWH. Third, five of six studies were only qualitative and included face-to-face interviews; social desirability biases may have played a role in some of the responses. Using computer-assisted quantitative surveys may offer additional privacy to respondents who may feel uncomfortable responding to sensitive HIV-related questions during an in-person interview.

## Conclusion

Overall, we noted a dearth of literature on HIV treatment and care for HLWH, much of which was qualitative. Despite sparse availability of literature, these findings suggested that the development of social and structural interventions that increase accessibility and acceptability of HIV care services and improve care outcomes for Hispanics/Latinas is vital. Reports of viral suppression rates of only 58% among Hispanics/Latinas underscore a need for improvement if we are to reach national goals of 80% of persons with HIV infection being virally suppressed. Researching and implementing effective strategies, including promoting active engagement and retention in care and follow-up of Hispanic/Latinas with missed HIV care visits, improving trust with culturally competent care, including Spanish-language staff persons and materials, improving provider engagement, and screening and finding solutions for support services, for example, mental health and violence situations, may be effective in increasing utilization of HIV care among HLWH and support the national plan to End the HIV Epidemic in the United States.^32^ At present, of 84 evidence-based HIV prevention interventions addressing care and medication adherence and strategies for persons with HIV infection,^3^ none was developed exclusively for Hispanics/Latinas. Future studies are needed to increase the numbers of interventions and inform best evidence-based practices for addressing social and structural factors that impede HIV care and treatment outcomes for HLWH in the United States.

## Abbreviations Used

ADAP: AIDS Drug Assistance program
ART: antiretroviral therapy
HLWH: Hispanics/Latinas with HIV
SDH: social determinants of health
